# Safety and Immunogenicity of MF59-Adjuvanted Cell Culture–Derived A/H5N1 Subunit Influenza Virus Vaccine: Dose-Finding Clinical Trials in Adults and the Elderly

**DOI:** 10.1093/ofid/ofz107

**Published:** 2019-03-01

**Authors:** Sharon E Frey, Sepehr Shakib, Pornthep Chanthavanich, Peter Richmond, Timothy Smith, Terapong Tantawichien, Claudia Kittel, Peter Jaehnig, Zenaida Mojares, Bikash Verma, Niranjan Kanesa-thasan, Matthew Hohenboken

**Affiliations:** 1 School of Medicine, Saint Louis University, St. Louis, Missouri; 2 CMAX Clinical Research Pty Ltd., Adelaide, SA, Australia; 3 Department of Tropical Pediatrics, Faculty of Tropical Medicine, Mahidol University, Bangkok, Thailand; 4 Division of Paediatrics, School of Medicine, University of Western Australia, and Vaccine Trials Group, Telethon Kids Institute, Subiaco, WA, Australia; 5 Mercy Health Research, St. Louis, Missouri; 6 Department of Medicine, Faculty of Medicine, Chulalongkorn University and Queen Saovabha Memorial Institute, Bangkok, Thailand; 7 GlaxoSmithKline Vaccines GmbH, Marburg, Germany; 8 GlaxoSmithKline Pte Ltd., Singapore, Singapore; 9 GlaxoSmithKline Vaccines LLC, Rockville, Maryland; 10 Seqirus Inc., Cambridge, Massachusetts

**Keywords:** cell culture–derived vaccine, H5N1 subunit vaccine, influenza, MF59 adjuvant; pandemic influenza, phase II

## Abstract

**Background:**

A/H5N1 influenza viruses have high pandemic potential; consequently, vaccines need to be produced rapidly. MF59® adjuvant reduces the antigen required per dose, allowing for dose sparing and more rapid vaccine availability.

**Methods:**

Two multicenter, phase II trials were conducted to evaluate the safety and immunogenicity of an MF59-adjuvanted, cell culture–derived, A/H5N1 vaccine (aH5N1c) among 979 adult (18–64 years old) and 1393 elderly (≥65 years old) subjects. Participants were equally randomized to receive 2 full-dose (7.5 μg of hemagglutinin antigen per dose) or 2 half-dose aH5N1c vaccinations 3 weeks apart. Outcomes were based on Center for Biologics Evaluation Research and Review (CBER) and Committee for Medicinal Products for Human Use (CHMP) licensure criteria (titers ≥1:40 and seroconversions on day 43). Solicited reactions and adverse events were assessed (www.clinicaltrials.gov: NCT01776541 and NCT01766921).

**Results:**

CBER and CHMP criteria were met by both age groups. CBER criteria for hemagglutination titers were met for the full-dose formulation. Solicited reaction frequencies tended to be higher in the full-dose group and were of mild to moderate intensity. No vaccine-related serious adverse events occurred.

**Conclusions:**

In adult and elderly participants, the full-dose aH5N1c vaccine formulation was well tolerated and met US and European licensure criteria for pandemic vaccines.

Periodic influenza pandemics pose serious threats to global health and economies [1]. The highly pathogenic avian influenza virus H5N1 causes severe human disease and death [1] and has a higher case fatality rate than seasonal influenza infections [1, 2]. Of the 694 H5N1 cases reported to the World Health Organization between 2003 and 2015, 402 (~58%) were fatal [3]. Sporadic cases of human H5N1 virus infection have been associated with close contact with infected poultry. Moreover, circulation of H5N1 virus in bird flocks allows for potential mutations that can facilitate bird-to-human and human-to-human viral transmission [1]. Overall, the H5N1 virus retains pandemic potential because it has spread to most continents, and most humans are unlikely to be immune to the virus [1].

Rapid and efficient production of pandemic influenza vaccines is essential to meet the anticipated global demand [4, 5]. New cell culture–based production methods can eliminate dependency on egg supply and poultry flocks, which are also vulnerable to H5N1 infection. Cell culture–based vaccine manufacturing techniques shorten production times and increase production capacity [4, 6].

Manufacturing capacity may also benefit from the use of a vaccine adjuvant to enhance immunogenicity. MF59^®^ (Novartis International AG, Basel, Switzerland) is a proprietary oil-in-water emulsion adjuvant that has been used in several registered pandemic and seasonal influenza vaccines since 1997. It has a well-established safety profile [7], allows for reduced antigen content per dose (7.5 μg of hemagglutinin [HA] in pandemic formulations vs 15 μg of HA per strain in seasonal formulations [6, 8–10]), promotes the production of cross-reactive antibodies that may provide heterologous immunity against antigenically divergent strains [8, 11, 12], and improves vaccine efficacy in elderly adults and children, who are particularly vulnerable to influenza infection [13].

Based on a previous phase I dose-ranging study of an adjuvanted, cell culture–derived, H5N1 subunit influenza virus vaccine, 2 antigen-sparing formulations were evaluated in phase II studies [14]. We present safety, tolerability, and immunogenicity data from healthy adult and elderly subjects to establish the optimal vaccine formulation for these age groups. Here we present primary and secondary outcome data from these 13-month studies, up to study day 43.

## METHODS

### Study Design

**Figure 1.**
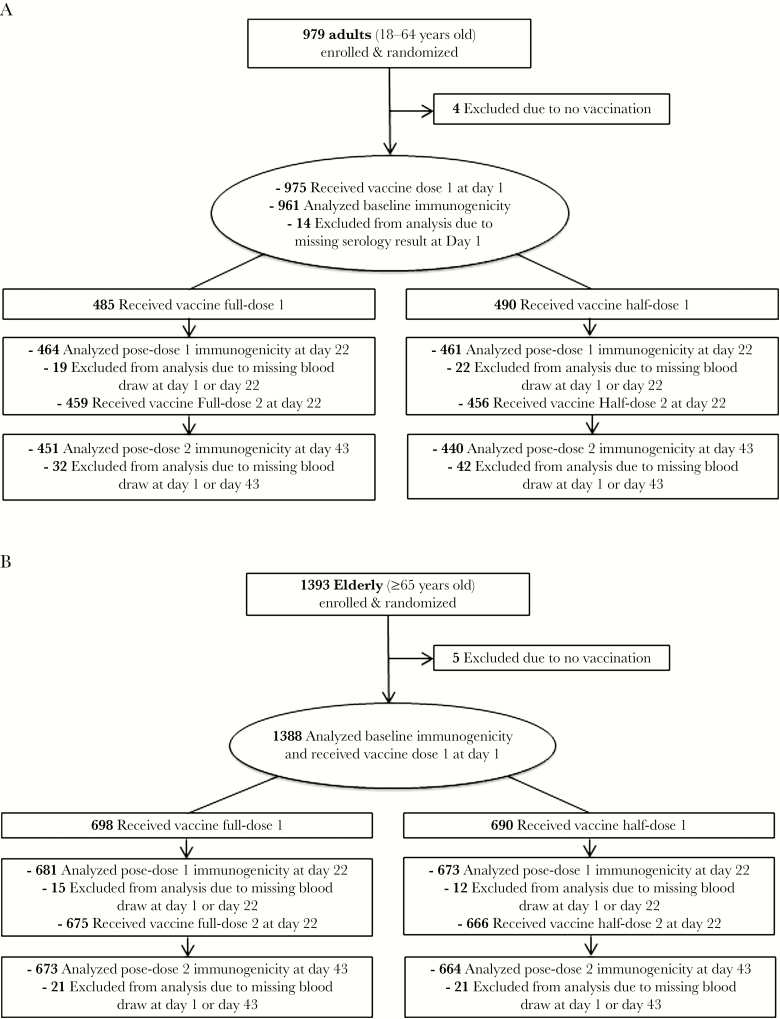
Study design and subject disposition for adult (A; NCT01776541) and elderly (B; NCT01766921) clinical trials.

The adult (NCT01776541) and elderly (NCT01766921) trials were phase II, randomized, observer-blind, multicenter studies, conducted in Australia, New Zealand, the United States, and Thailand. The design, objectives, and endpoints were identical for both studies. The study protocols were approved by the Ethics Review Committees of the participating centers, and the studies were conducted in compliance with Good Clinical Practices guidelines and the Declaration of Helsinki. Written informed consent was obtained from subjects before enrollment. Subjects were randomized at a 1:1 ratio to receive 2 vaccinations with either full-dose or half-dose MF59-adjuvant, cell culture–derived, H5N1 vaccine (aH5N1c) given 3 weeks apart (Figure 1). The primary immunogenicity outcomes evaluated hemagglutination inhibition (HI) assay antibody responses in terms of the percentages of subjects achieving seroconversion and HI titers ≥1:40 on day 43. Each subject was followed for 12 months (day 387) after the second vaccine dose to assess safety and immunogenicity.

### Study Participants

In the respective studies, 979 adult (aged 18–64 years) and 1393 elderly subjects (aged ≥65 years) were enrolled. The main exclusion criteria were presence of serious chronic or progressive disease, pregnancy or breastfeeding, prior receipt of any H5N1 vaccine, receipt of any other influenza vaccines within 60 days before enrollment, body temperature ≥38.0°C and/or any acute illness within 3 days of receiving study vaccines, and a body mass index ≥35 kg/m^2^ (see ClinicalTrials.gov for all exclusion criteria).

### Vaccines

Both the adult and elderly studies used MF59-adjuvanted, cell culture–derived, monovalent, inactivated, subunit, H5N1 vaccines containing A/turkey/Turkey/1/05 (H5N1)-like strain (NIBRG-23) antigen (Seqirus Inc., Holly Springs, NC; *f/k/a* Novartis Influenza Vaccines GmbH, Marburg, Germany). One 0.5 mL dose (full-dose formulation) contained 7.5 μg of HA antigen with 0.25 mL of MF59. One 0.25 mL dose (half-dose formulation) contained 3.75 μg of HA with 0.125 mL of MF59. Vaccines were administered on day 1 and day 22 as single intramuscular injections in the nondominant arm.

### Immunogenicity Assessments

Sera samples were obtained for immunogenicity analyses before each vaccination (day 1 and day 22) and on day 43 and day 387 (stored at –18°C). Immunogenicity was assessed by HI assay against the H5N1 vaccine strain according to standard methods [15] and expressed as the percentage of subjects achieving seroconversion, the percentage of subjects with an HI titer ≥1:40, geometric mean HI titers (GMTs), and the geometric mean ratios (GMRs) of HI titers. Seroconversion was defined as HI titers ≥1:40 for subjects who were seronegative at baseline (HI titer <1:10 on day 1) or a minimum 4-fold increase in HI titer for subjects who had detectable baseline HI titers (≥1:10). For both studies, the primary immunogenicity outcomes were assessed on day 43 and were based on the licensure criteria for pandemic influenza vaccines established by the Center for Biologics Evaluation Research and Review (CBER) [16]. For the adult population, the following CBER criteria were applied: (1) the lower limit (LL) of the 2-sided 97.5% confidence interval (CI) for the percentage of subjects achieving seroconversion for HI antibody responses should be ≥40%; (2) the LL of the 2-sided 97.5% CI for the percentage of subjects achieving an HI antibody titer of ≥1:40 should be ≥70%. For the elderly population, the values for the criteria described above were (1) ≥30% and (2) ≥60%, respectively. The secondary immunogenicity outcomes were also assessed on day 43 and were based on the licensure criteria for pandemic influenza vaccines established by the Committee for Medicinal Products for Human Use (CHMP) [17]. For the adult population, the following CHMP criteria applied: 1) the LL of the 2-sided 97.5% CI for the percentage of subjects achieving seroconversion for HI antibody responses should be ≥40%; 2) the LL of the 2-sided 97.5% CI for the percentage of subjects achieving an HI antibody titer of ≥1:40 should be ≥70%; 3) GMR should be >2.5. For the elderly population, the values for the criteria described above were (1) ≥30%, (2) ≥60%, and (3) >2.0, respectively.

### Safety Assessments

After each vaccination, subjects were observed for 30 minutes to monitor for immediate reactions. Solicited local and systemic reactions were recorded by the subjects on diary cards for 7 days after each vaccination. Solicited local reactions included injection site induration, erythema, ecchymosis, and pain. Solicited systemic reactions included nausea, generalized myalgia, generalized arthralgia, headache, fatigue, loss of appetite, malaise, and fever (body temperature ≥38°C). The severity of solicited reactions was categorized as mild (transient with no limitation in normal daily activity), moderate (some limitation in normal daily activity), or severe (unable to perform normal daily activity). All unsolicited adverse events (AEs) were collected for 21 days after each vaccination. Serious adverse events (SAEs), the new onset of chronic diseases, medically attended AEs, AEs of special interest, AEs leading to study withdrawal, and the administration of concomitant medications associated with these events were recorded throughout the study. The causal relationships of AEs to the study vaccines were assessed by the investigators as being either nonrelated, possibly related, or probably related.

### Statistical Analyses

Sample sizes for the adult and elderly studies were planned as 486 and 624 evaluable subjects per group (assuming a study dropout rate of 10%), respectively. The percentages of subjects achieving seroconversion and the percentages of subjects with HI titers ≥1:40, along with the associated 97.5% Clopper-Pearson CIs, were calculated as log_10_-transformed values using analysis of covariance (ANCOVA) with factors for dose, group, baseline titer, and study center. GMTs, GMRs, and the associated 2-sided adjusted 95% CIs were calculated using ANCOVA with factors for race, gender, and study center. Although the CBER criteria involve the lower limits of 95% CIs, a 97.5% CI was calculated because there were 2 vaccine formulations tested in the study, and the 0.05 alpha was distributed across tests. The immunogenicity analyses were performed on full analysis set (FAS) data, which included all subjects who received at least 1 dose of study vaccine and provided at least 1 serum sample at both prevaccination and postvaccination time points. Analyses of vaccine reactogenicity and safety were performed on data from subjects who had received at least 1 study vaccination and provided either postvaccination AE or reactogenicity data (safety data set). All safety analyses were descriptive.

## RESULTS

Of the enrolled 979 adult and 1393 elderly subjects, 975 (>99%) and 1388 (>99%) received at least 1 dose of study vaccine, respectively (Figure 1). Overall, 91% of adult subjects (451 of 488 in the full-dose group and 440 of 491 in the half-dose group) and 96% of elderly subjects (673 of 700 in the full-dose group and 664 of 693 in the half-dose group) remained in the study and provided sera for immunogenicity analyses on day 43 (FAS data) (Figure 1). Approximately 9% of adult subjects and 4% of elderly subjects were excluded from the immunogenicity FAS data due to the absence of sera data. Subject demographics and baseline characteristics were well balanced between groups within the respective study populations (Table 1). In both studies, more females were enrolled than males (56% vs 44% in the adult study and 59% vs 41% in the elderly study, respectively).

**Table 1.** Study Population Demographics

	Adult Subjects		Elderly Subjects	
	(Age 18–64 y, n = 979)		(Age ≥65 y, n = 1393)	
	Full-Dose	Half-Dose	Full-Dose	Half-Dose
	(n = 488)	(n = 491)	(n = 700)	(n = 693)
Age, mean ± SD, y	39.0 ± 13.7	38.4 ± 14.2	71.2 ± 5.1	70.7 ± 4.7
Male, No. (%)	203 (42)	232 (47)	293 (42)	275 (40)
Weight, mean ± SD, kg	74.1 ± 15.1	73.5 ± 16.3	71.0 ± 15.9	71.4 ± 16.2
Height, mean ± SD, cm	167.8 ± 10.5	168.5 ± 11.0	164.1 ± 10.7	163.5 ± 10.9
BMI, mean ± SD, kg/m^2^	26.2 ± 4.3	25.7 ± 4.3	26.1 ± 4.0	26.5 ± 4.2
Previous influenza vaccination, No. (%)	118 (24)	119 (24)	429 (61)	419 (60)
Influenza vaccination ≤12 mo,^a^ No. (%)	98 (20)	95 (19)	146 (21)	137 (20)
White, No. (%)	291 (60)	290 (59)	445 (64)	444 (64)
Asian, No. (%)	93 (19)	96 (20)	240 (34)	237 (34)
Black/African American, No. (%)	97 (20)	99 (20)	10 (1)	10 (1)
American Indian/Alaska Native, No. (%)	2 (<1)	1 (<1)	2 (<1)	0
Native Hawaiian/Pacific Islander, No. (%)	0	1 (<1)	0	0

Full-dose: 7.5 µg of aH5N1c antigen + 0.25 mL of MF59 adjuvant per dose; Half-dose: 3.75 µg of aH5N1c antigen + 0.125 mL of MF59 adjuvant per dose.

Abbreviation: BMI, body mass index; SD, standard deviation.

^a^Twelve months before enrollment in the study.

### Immunogenicity

At day 43, 83% (97.5% CI, 78%–87%) and 61% (97.5% CI, 56%–66%) of adult subjects in the full-dose and half-dose groups, respectively, achieved seroconversion; the CBER and CHMP criteria for seroconversion were met for both treatment groups (Table 2) [16, 17]. In the elderly population, 74% (97.5% CI, 70%–77%) and 52% (97.5% CI, 48%–56%) of subjects in the full-dose and half-dose groups achieved seroconversion at day 43, respectively; the CBER and CHMP criteria for seroconversion were met for both treatment groups.

**Table 2.** Percentage of Subjects With HI Titers ≥1:40 at Day 1 and Day 43, GMRs Day 43/Day 1, and Percentages of Subjects Achieving Seroconversion or Significant Increases in HI Titers at Day 43

	Adult Subjects		Elderly Subjects	
	(Age 18–64 y, n = 891)		(Age ≥65 y, n = 1337)	
	Full-Dose	Half-Dose	Full-Dose	Half-Dose
	(n = 451)	(n = 440)	(n = 673)	(n = 664)
Day 1: HI titers ≥1:40 (97.5% CI), %	4.0 (2.0–7.0)	4.0 (2.0–6.0)	12 (10–15)	10 (8.0–13)
Day 43: HI titers ≥1:40 (97.5% CI), %	85 (81–88)^a,b^	63 (58–68)	81 (77–84)^a,b^	63 (58–67)
Day 43/day 1: GMRs (95% CI),^c^	41 (34–49)^b^	11 (8.7–13)^b^	16 (14–18)^b^	5.7 (5.0–6.6)^b^
Day 43: Positivity conversion (97.5% CI), %	83 (78–87)	61 (55–67)	76 (71–80)	56 (51–61)
Day 43: Significant increase (97.5% CI), %	83 (69–92)	61 (43–77)	66 (57–74)	38 (29–47)
Day 43: Seroconversion (97.5% CI), %	83 (78–87)^a,b^	61 (56–66)^a,b^	74 (70–77)^a,b^	52 (48–56)^a,b^

Abbreviations: CI, confidence interval; GMR, geometric mean ratio; HI, hemagglutination inhibition; seroconversion, defined as positivity or significant increase.

^a^Outcome met CBER criterion [16] for age group.

^b^Outcome met CHMP criterion [17] for age group.

^c^GMR was a secondary end point in the adult study and was not adjusted for multiplicity; therefore, GMR (97.5% CI) data are not available. Positivity conversion was defined as postvaccination HI titer ≥1:40 for subjects negative (titer < 1:10) at baseline or a minimum 4-fold increase in HI titer for subjects seropositive (titer ≥ 1:10) at baseline; significant increase in antibody titer was defined as a minimum 4-fold increase in HI titer for subjects seropositive (titer ≥ 1:10) at baseline.

At day 43, 85% (97.5% CI, 81%–88%) and 63% (97.5% CI, 58%–68%) of adult subjects in the full-dose and half-dose groups achieved HI titers ≥1:40, respectively (Table 2). The LL of the 2-sided 97.5% CI for the percentage of subjects achieving an HI antibody titer ≥1:40 exceeded the CBER and CHMP requirements of 70% in the full-dose group, but not in the half-dose group. In the elderly population, 81% (97.5% CI, 77%–84%) of subjects in full-dose group and 63% (97.5% CI, 58%–67%) of subjects in the half-dose group achieved HI antibody titers ≥1:40 at day 43. The LL of the 2-sided 97.5% CI exceeded the CBER and CHMP criterion of 60% in the full-dose group, but not in the half-dose group.

**Figure 2.**
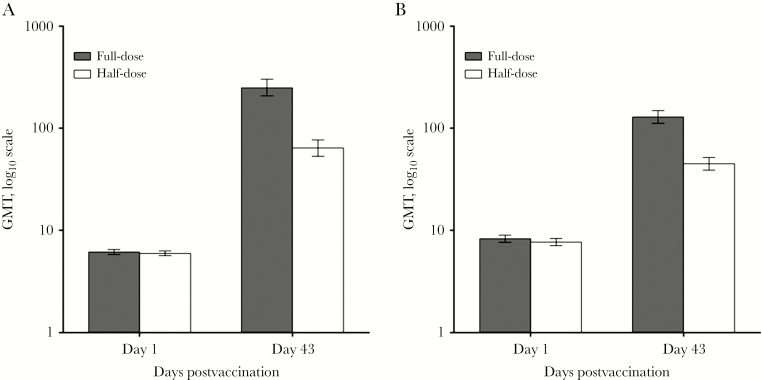
Geometric mean hemagglutination inhibition antibody titers (95% confidence interval) on day 1 and day 43 in adult (A; age 18–64 years) and elderly (B; age ≥65 years) subjects.

At baseline, GMTs within each study population were comparably low between the respective vaccine groups. Among adult subjects, 3 weeks after 2 doses of aH5N1c (day 43), GMTs rose to 250 (95% CI, 208%–302%) and 64 (95% CI, 53%–77%) in the full-dose and half-dose groups, respectively (Figure 2A). The day 43 to day 1 GMRs for the full-dose and half-dose groups were 41 (95% CI, 34%–49%) and 11 (95% CI, 9%–13%), respectively, and exceeded the CHMP GMR criterion of 2.5. Among elderly subjects, GMTs rose to 129 (95% CI, 112%–149%) and 45 (95% CI, 39%–52%) by day 43 in the full-dose and half-dose groups, respectively (Figure 2B). The day 43 to day 1 GMRs for the elderly full-dose and half-dose groups were 16 (95% CI, 14%–18%) and 5.7 (95% CI, 5%–7%), respectively, exceeding the CHMP criterion of 2.0 (Table 2).

### Safety

The rates of any solicited reactions within 30 minutes of administration of the first vaccine dose were low and comparable between the vaccine groups in each age population, with no increase in reactogenicity after administration of the second dose (adults 7% vs 5% following first and second doses; elderly 8% vs 7% following first and second doses, respectively). In both age populations, the frequencies of solicited local reactions reported within 7 days of each vaccination were higher for the full-dose groups compared with the respective half-dose groups, with no increase in reactogenicity after administration of the second doses (Table 3). For both age populations, injection site pain was the most frequently reported solicited local reaction. Within each age population, frequencies of individual solicited systemic reactions reported within 7 days of each vaccination were comparable between the respective full-dose and half-dose groups (Table 3). Among adults, after each vaccination, the most commonly reported systemic reactions were headache and fatigue, followed by malaise, with no difference between the full-dose and half-dose groups. Among elderly subjects, the most commonly reported systemic reactions after each vaccination were fatigue and malaise, with no difference between the full-dose and half-dose groups. After the second vaccination, frequencies of solicited systemic reactions were lower.

**Table 3.** Overview of Solicited Adverse Events and Other Indicators of Reactogenicity During a 7-Day Period After Vaccination

	Adult Subjects				Elderly Subjects			
	(Age 18–64 y, n = 944)				(Age ≥65 y, n = 1376)			
	First Vaccination		Second Vaccination		First Vaccination		Second Vaccination	
	Full-Dose	Half-Dose	Full-Dose	Half-Dose	Full-Dose	Half-Dose	Full-Dose	Half-Dose
	(n = 468)	(n = 469)	(n = 450)	(n = 443)	(n = 692)	(n = 681)	(n = 676)	(n = 665)
Any reaction, %	72	56	53	43	52	39	39	28
Local reactions, No. of subjects	460–462	464–466	445–448	439–442	677–685	667–673	672–674	660–662
Local reactions, %	63	43	48	34	38	22	29	17
Pain (% severe), %	63 (<1)	43 (1)	48 (0)	34 (<1)	38 (0)	21 (0)	29 (<1)	16 (<1)
Ecchymosis (% severe), %	1 (0)	<1 (0)	1 (0)	1 (0)	<1 (0)	1 (0)	1 (0)	1 (0)
Erythema (% severe), %	1 (0)	0	0	0	2 (0)	<1 (0)	1 (0)	<1 (0)
Induration (% severe), %	3 (<1)	1 (0)	1 (0)	<1 (0)	2 (0)	1 (0)	2 (0)	<1 (0)
Systemic reactions, No. of subjects	452–457	452–460	439–446	432–438	668–688	656–677	663–676	652–664
Systemic reactions, %	43	38	27	23	28	26	19	17
Fatigue (% severe), %	23 (1)	19 (1)	13 (<1)	11 (0)	12 (<1)	11 (<1)	9 (<1)	9 (<1)
Nausea (% severe), %	7 (1)	8 (<1)	6 (<1)	4 (0)	4 (<1)	4 (<1)	3 (0)	3 (<1)
Malaise (% severe), %	21 (2)	16 (1)	12 (<1)	9 (0)	12 (<1)	12 (<1)	8 (<1)	8 (<1)
Myalgia (% severe), %	19 (1)	14 (<1)	10 (0)	8 (0)	10 (0)	10 (0)	7 (<1)	5 (0)
Arthralgia (% severe), %	12 (1)	9 (1)	6 (0)	5 (0)	6 (<1)	8 (<1)	5 (<1)	4 (<1)
Headache (% severe), %	21 (1)	20 (<1)	14 (<1)	11 (0)	10 (<1)	10 (<1)	6 (<1)	6 (0)
Loss of appetite (% severe), %	8 (<1)	7 (<1)	5 (<1)	3 (0)	4 (0)	5 (<1)	4 (0)	2 (0)
Temperature, % ≥38°C (% ≥40°C)	2 (<1)	2 (0)	1 (0)	<1 (0)	2 (0)	<1 (0)	1 (0)	<1 (0)
Other reactions, No. of subjects	458–465	456–462	434–449	429–438	678–688	671–677	673–676	657–664
Prevention of pain/fever (% severe), %	3 (1)	8 (2)	4 (1)	6 (1)	10 (1)	18 (3)	11 (2)	8 (1)
Treatment of pain/fever (% severe), %	34 (7)	24 (5)	15 (3)	7 (2)	36 (5)	29 (4)	23 (3)	19 (3)

Between day 1 and day 43, unsolicited AEs were reported by up to 29% of subjects in the adult population and up to 32% of subjects in the elderly population, with comparable frequencies between the vaccine groups in each study. Following first or second vaccinations, 10% and 12% of subjects in the adult and elderly populations reported an unsolicited AE that was judged to be possibly or probably related to the study vaccine, respectively. Injection site bruising was the most commonly reported unsolicited AE in both age groups. At least 1 SAE was reported by 3 (<1%) subjects (appendicitis, pyelonephritis, and nerve compression) in the adult population, and by 10 (1%) subjects (atrial fibrillation, adenocarcinoma of left lung, transient ischemic attack, cholecystitis, benign positional vertigo, right inguinal hernia, acute kidney injury, infected wound of left leg, fractured ribs, and syncope) in the elderly population; none of the reported SAEs were considered to be possibly or probably vaccine-related. One elderly subject (<1%) withdrew prematurely from the study due to an unsolicited AE. Three (<1%) subjects in the adult population and 24 (2%) subjects in the elderly population were diagnosed with the new onset of chronic diseases up to day 43. Only 1 SAE had a fatal outcome (non-vaccine-related, elderly half-dose group; lung adenocarcinoma; occurred on day 155).

## Discussion

We evaluated the immunogenicity and safety of an MF59-adjuvanted, cell culture–derived, H5N1 subunit influenza virus vaccine in 2 phase II studies; vaccine was administered as either a full- or half-dose formulation to healthy adult or elderly subjects. For both age populations, all CBER criteria (seroconversion and HI titer ≥1:40) were met for the full-dose group, whereas the half-dose group only met the criteria for seroconversion. Similarly, for both age groups, all CHMP criteria (seroconversion, HI titer ≥1:40, and GMR) were met for the full-dose group, whereas the half-dose group met 2 of the 3 criteria (seroconversion and GMR). Although both vaccine formulations were immunogenic and well tolerated in each age population, the full-dose (7.5 μg of H5N1 HA antigen with 0.25 mL of MF59) aH5N1c vaccine appears preferable—in terms of higher immunogenicity—for future clinical development. Overall, postvaccination immunogenicity outcomes (seroconversion, percentages of subjects with HI titers ≥1:40, and GMTs) were higher among adults compared with elderly subjects, regardless of vaccine formulation. The generally lower postvaccination antibody responses observed in the elderly population may partly be attributed to immunosenescence.

In both studies, the proportions of subjects who reported solicited reactions trended higher for the full-dose group compared with the half-dose group (mainly due to incidence of pain at the site of injection). Subjects ≥65 years of age appeared to report fewer solicited local and systemic reactions in each vaccine group compared with adult subjects. Irrespective of vaccine formulation, and for each age group, the majority of reported reactions were mild to moderate in severity, with no evidence of increased frequency of reactions following a second dose. In both age groups, there were no appreciable differences in unsolicited AEs between the full-dose and half-dose groups, in terms of both frequencies and specific conditions.

The immunogenicity data for the full-dose formulation are consistent with those of previous studies conducted in healthy adult and elderly individuals, in which subjects received 2 doses of MF59-adjuvanted H5N1 vaccine (7.5 μg of H5N1 HA per dose) [8, 11, 14, 18, 19]. Whereas in the present study the half-dose formulation (containing 3.75 μg of H5N1 antigen and a lower-than-standard quantity of MF59 per dose) did not meet all the licensure criteria evaluated, many studies conducted to assess the immunogenicity of 2 half-doses (3.75 μg of antigen with a reduced quantity of MF59) administered 3 weeks apart have consistently demonstrated that either 1 or 2 half-doses were sufficiently immunogenic to meet the CBER and/or CHMP licensure criteria; these studies included subjects of all ages and ethnicities, cell- and egg-derived vaccines, and A/H5N1 [14], A/H3N2 [20], and A/H1N1 [6, 21–28] strains. Overall, the reactogenicity and safety data presented here are similar to and are in agreement with the results of previous studies of MF59-adjuvanted H5N1 vaccine when administered to healthy adult and elderly subjects [8, 19].

There were some limitations to the study. First, because a non-H5N1 control group was not included, interpretation of the safety data is somewhat limited. However, the safety profile of the full-dose formulation observed in this study is consistent with that reported from a recent placebo-controlled trial of an egg-based, MF59-adjuvanted, H5N1 vaccine (Aflunov, Seqirus Vaccines Ltd., Liverpool, UK [*f/k/a* Novartis Influenza Vaccines Ltd., Liverpool, UK]; 7.5 μg of H5N1 HA per dose) conducted in adult and elderly subjects [19]. Second, the study assessed only short-term antibody responses; analyses of mid-term (6-month) responses and long-term antibody persistence are either ongoing or planned. Third, the relatively short follow-up period does not allow for the possible detection of rare, delayed AEs. Nonetheless, in a separate study of a cell culture–derived, MF59-adjuvanted H5N1 vaccine in adults, no vaccine-related AEs were reported throughout the study period [14].

In conclusion, two 7.5-μg doses of a cell culture–derived, MF59-adjuvanted H5N1 vaccine administered 3 weeks apart were well tolerated and highly immunogenic, raised no safety concerns, and induced robust antibody responses in adult and elderly subjects that met all the immunogenicity criteria required for pandemic vaccine licensure by both the US and European regulatory authorities. These results support the further development of the full-dose (7.5 μg), MF59-adjuvant, cell culture–derived H5N1 vaccine formulation for adult (age ≥18 years) pandemic preparedness programs.
